# Catheter ablation in patients with atrial fibrillation and dilated cardiomyopathy

**DOI:** 10.3389/fcvm.2024.1305485

**Published:** 2024-01-16

**Authors:** Yoon-Kee Siow, Chin-Yu Lin, Fa-Po Chung, Yenn-Jiang Lin, Shih-Lin Chang, Li-Wei Lo, Yu-Feng Hu, Jo-Nan Liao, Ting-Yung Chang, Ta-Chuan Tuan, Ling Kuo, Cheng-I Wu, Chih-Min Liu, Shin-Huei Liu, Guan-Yi Li, Ming-Jen Kuo, Shang-Ju Wu, Jose Antonio Bautista, Yu-Shan Huang, Dinh Son Ngoc Nguyen, Shih-Ann Chen

**Affiliations:** ^1^ Heart Rhythm Center, Taipei Veterans General Hospital, Taipei, Taiwan; ^2^ Division of Cardiology, Serdang Hospital, Selangor, Malaysia; ^3^ Department of Medicine, National Yang Ming Chiao Tung University, Taipei, Taiwan; ^4^ Cardiovascular Center, Taichung Veterans General Hospital, Taichung, Taiwan; ^5^ Department of Medicine, National Chung Hsing University, Taichung, Taiwan

**Keywords:** atrial fibrillation, dilated cardiomyopathy, catheter ablation, mortality, clinical outcomes

## Abstract

**Introduction:**

Catheter ablation is an effective and safe strategy for treating atrial fibrillation patients. Nevertheless, studies on the long-term outcomes of catheter ablation in patients with dilated cardiomyopathy are limited. This study aimed to assess the electrophysiological characteristics of atrial fibrillation patients with dilated cardiomyopathy and compare the long-term clinical outcomes between patients undergoing catheter ablation and medical therapy.

**Method:**

Patient baseline characteristics and electrophysiological parameters were examined to identify the predictors of atrial fibrillation recurrence following catheter ablation. The clinical outcomes of catheter ablation and medical therapy were compared using the propensity score matched method.

**Results:**

A total of 343 patients were enrolled, with 46 in the catheter ablation group and 297 in the medical therapy group. Among the catheter ablation group, 58.7% (*n* = 27) had persistent atrial fibrillation. The recurrence rate of atrial arrhythmia was 30.4% (*n* = 14) after an average follow-up duration of 7.7 years following catheter ablation. The only predictive factor for atrial fibrillation recurrence after catheter ablation was the left atrial diameter. When compared to medical therapy, catheter ablation demonstrated significantly better outcomes in terms of overall survival, freedom from heart failure hospitalization, improvement in left ventricular ejection fraction, and a greater reduction in left ventricular diameter and left atrial diameter after propensity score matching.

**Conclusions:**

Therefore, catheter ablation proves to be effective in providing long-term control of atrial fibrillation in patients with dilated cardiomyopathy. In addition to standard heart failure care, catheter ablation significantly enhanced both morbidity and mortality outcomes and reversed structural remodeling when compared to heart failure medication alone.

## 1 Introduction

Atrial fibrillation (AF) is one of the most common arrhythmias that predisposes patients to a higher risk of morbidity and mortality. The prevalence of AF is approximately 1% in the United States (1), while it is estimated to be 0.6%–1.5% in the Asian population (2–4).

Dilated cardiomyopathy (DCM) is defined as a dilated left ventricular (LV) chamber with a poor left ventricular ejection fraction (LVEF) in the absence of coronary artery disease, hypertension, valvular disease, or congenital heart disease (5). DCM is a severe cardiac disorder in which structural or functional abnormalities of the heart muscle can lead to substantial morbidity and mortality due to complications such as heart failure (HF) and arrhythmia (6).

AF and HF often coexist, while HF precedes AF leading to a worse outcome (7). When cardiomyopathy and AF occur concurrently, patients may experience worse symptoms and poorer prognosis (8). Previous studies such as AATAC and Castle-AF showed that catheter ablation (CA) is superior to medical therapy (9, 10). A recently published Castle-HTx demonstrated that the combination of CA and guideline-directed medical therapy (GDMT) improves survival in end-stage HF patients (11). However, evidence-based evaluations and previous studies did not specifically address this complex subgroup of patients with AF, poor LVEF, and a dilated LV chamber.

We hypothesized that a vicious cycle exists between AF and LV dysfunction in patients with DCM. AF results in atrial stunning and arrhythmia-induced cardiomyopathy, leading to the deterioration of preexisting LV dysfunction and chamber dilatation (8). The LV dysfunction increases the left atrium (LA) pressure, which causes LA dilatation and AF (12). Based on this theory, CA may provide better outcomes in terms of AF control and the reversal of LV dysfunction and structural remodeling compared to medical therapy. Therefore, this study aimed to examine the long-term clinical outcomes of CA in patients with AF and DCM and compare these outcomes with those of patients who underwent medical therapy without CA.

## 2 Method

### 2.1 Study population

This retrospective cohort study included consecutive patients with documented DCM and AF who underwent CA for drug-refractory AF (Group 1) and those who did not undergo CA (Group 2) between January 2001 and November 2021 at Taipei Veterans General Hospital. Data were analyzed between November 1, 2022 and March 31, 2023. The baseline characteristics, echocardiographic parameters, and electrophysiological findings of the patients were assessed. DCM was defined as an LVEF less than 45% and a left ventricular internal dimension in diastole (LVIDd) of more than 5.3 cm for females and 5.9 cm for males, respectively (13). AF was defined based on electrocardiography (ECG) or Holter monitoring. Paroxysmal and persistent AF were defined according to an updated consensus (14).

This study was approved by the Institutional Ethics Review Board of the Taipei Veterans General Hospital (IRB No. 2021-11-015BC). Given the retrospective nature of this study, the requirement for informed consent was waived by the institutional review board.

### 2.2 Inclusion and exclusion criteria

Patients diagnosed with concomitant DCM and AF were included. Exclusions were made for patients with secondary causes of DCM, such as hypertension, coronary heart disease, congenital heart disease, valvular heart disease, peripartum conditions, alcoholic and metabolic diseases like thyroid disease. Additionally, patients with a history of previous cardiovascular surgeries, Cox maze procedures, in-hospital and out-of-hospital cardiac arrests, a life expectancy of less than one year after diagnosis, and incomplete data were excluded from this study. Patients suspected of arrhythmia-induced cardiomyopathy, showing improved LVEF after rate control were also excluded from this study.

#### 2.3.1 Part I: ablation procedure

The protocols for the CA procedures have been described in our previous studies (15–18). Anti-arrhythmic medications were discontinued for at least five half-lives before the procedure. A 7F decapolar catheter with a 2-mm interelectrode distance and 5-mm spacing between each electrode pair was introduced into the coronary sinus. Transseptal atrial punctures were performed using fluoroscopic landmarks or transesophageal echocardiography and an 8.5F SL-0 sheath was inserted into the left atrium.

In patients with paroxysmal AF, we performed wide antral pulmonary vein isolation (PVI). In patients with non-paroxysmal AF, the ablation approach was described in the previous studies (18). Briefly, if sinus rhythm was not restored after PVI, linear ablation (mitral or roof ablation) was performed in selected patients with documented atypical flutter. Substrate modification was performed when non-pulmonary vein (PV) triggers were identified (17, 19, 20). Cardioversion was performed in cases in which all the above procedures failed to restore sinus rhythm.

Non-PV triggers were mapped and ablated (16). The strategy described earlier was applied to the atrial tachycardia (AT), which lasted for more than one minute. Subsequently, induction of AF was performed. If left atrial flutter or AT was induced and sustained for more than one minute, a re-entry circuit was identified, followed by additional focal or linear ablation.

Cavotricuspid isthmus (CTI) ablation was performed at the end of the procedure in patients with documented or inducible atrial flutter. Bidirectional blocks were achieved during the sinus rhythm.

#### 2.3.2 Part I: follow-up and detection of arrhythmia recurrence after ablation in group 1

After the procedure, patients were scheduled to visit the outpatient clinic for regular follow-up (two weeks, then every 1–3 months after CA). Routine ECG was performed for each outpatient follow-up, and 24-h Holter monitoring was performed at 3, 6, and 12 months. In addition, for patients experiencing symptoms suggestive of recurrence, 24-h Holter monitoring or cardiac event recording was performed to ascertain the types of arrhythmias.

#### 2.3.3 Part II: data collection for the clinical outcome for overall patients

Patients in Group 2 were followed up in the outpatient clinic for HF care (every 2–3 months after the first presentation). Data on patient baseline characteristics, medication history, electrophysiological features, and clinical outcomes, including major adverse cardiovascular events (MACE), changes in LVEF, left atrial diameter (LAD), and HF events, were collected.

The primary outcome was all-cause mortality. The secondary outcomes included cardiovascular mortality, ischemic stroke, myocardial infarction, hospitalization due to HF, changes of mean heart rate, LVEF, LVIDd and LAD. The cardiovascular mortality in the secondary outcome included composite death of acute coronary syndrome, myocardial infarction and heart failure. The mean heart rate was determined by resting heart rate recorded during two consecutive outpatient clinic visits.

The primary and secondary outcomes were identified through the hospital electronic database. The nationally linked electronic database was utilized to ascertain the cause of death for each patient. Phone calls to patients were also conducted to detect events that occurred in other hospitals.

To minimize the impact of confounding factors on clinical characteristics, we employed propensity analysis and matching techniques. We matched one-to-one pairs (Group 1 vs. Group 2) with identical propensity scores (PS) and a 0.01 calliper width. Suitability was assessed by estimating the standardized differences between the two groups in baseline characteristics (age, sex, and heart rate), comorbidities, echocardiographic parameters (LVEF, LVIDd, and LAD), and medications (oral anticoagulant and HF medication).

### 2.4 Statistical analysis

Categorical variables were presented as frequencies and percentages and compared using the chi-square and Fisher's exact tests. The Student's *t*-test was used to analyze normally distributed data. Continuous variables are presented as mean ± standard deviation and compared using the Mann–Whitney *U* test, as the data distribution was not normal. Univariate Cox regression analysis was initially performed. Differences in study endpoints were analyzed using the Kaplan–Meier method with the log-rank test. A *p*-value of <.05 with a 95% confidence interval was considered statistically significant. All statistical tests were performed using the IBM SPSS Statistics software version 26 (SPSS, Inc., Chicago, IL).

## 3 Results

**Figure 1:**
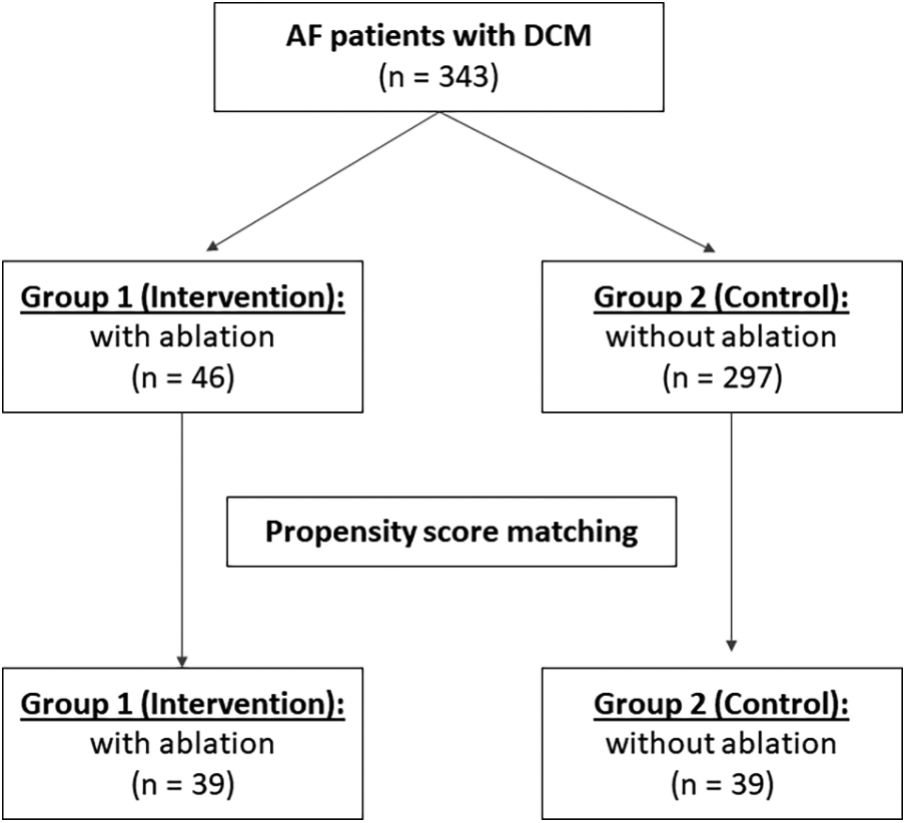
Patient flow chart. AF indicates atrial fibrillation; DCM, dilated cardiomyopathy.

A total of 8,507 patients with cardiomyopathy were screened. 343 patients (4.0%) with DCM and concomitant AF were recruited for this study, with 46 patients (13.4%) receiving CA in Group 1 and 297 patients (86.6%) receiving medical therapy in Group 2 (Figure 1).

### 3.1 Part I study

#### 3.1.1 Baseline characteristics and electrophysiological characteristics

The baseline and electrophysiological characteristics of the patients in Group 1 are shown in Table 1. The mean age was 59.3 ± 10.0 years, with 87.0% (*n* = 40) male, and the mean heart rate was 88.4 ± 25.8 beats per minute. A total of 8.7% (*n* = 4) of Group 1 patients were diagnosed with diabetes. The mean CHA_2_DS_2_VASc score was 2.0 ± 1.0. Twenty-seven patients (58.7%) had persistent AF. The mean LVEF was 37.0 ± 7.1%, the mean LVIDd was 60.6 ± 5.0 mm, and the mean LAD was 45.1 ± 6.0 mm.

**Table 1:** Baseline characteristics, comorbidities, echocardiographic parameters and medications before and after propensity score matching.

	Before PSM, *n* (%)			*p* value	After PSM, *n* (%)			*p* value
	Total (*n* = 343)	Group 1 (Intervention) (*n* = 46)	Group 2 (Control) (*n* = 297)		Total (*n* = 78)	Group 1 (Intervention) (*n* = 39)	Group 2 (Control) (*n* = 39)	
Baseline characteristics								
Age^a^ (mean ± SD)	71.5 ± 14.7	59.3 ± 10.0	73.4 ± 14.4	<0.001**	63.1 ± 12.3	61.3 ± 8.9	65.0 ± 14.8	0.667
Sex (Male)	241 (70.3%)	40 (87.0%)	201 (67.7%)	0.013*	60 (76.9%)	33 (84.6%)	27 (69.2%)	0.179
Heart rate^a^, bpm (mean ± SD)	90.2 ± 22.2	88.4 ± 25.8	90.5 ± 21.7	0.528	88.0 ± 24.9	88.0 ± 26.9	88.1 ± 23.0	0.893
Comorbidities								
Diabetes mellitus	99 (28.7%)	4 (8.7%)	95 (32.0%)	<0.001**	9 (11.5%)	4 (10.3%)	5 (12.8%)	0.999
CHA_2_DS_2_VAS_c_^a^ (mean ± SD)	3.6 ± 1.7	2.0 ± 1.0	3.8 ± 1.7	<0.001**	2.2 ± 1.2	2.1 ± 1.0	2.3 ± 1.4	0.999
Types of AF								
Paroxysmal	180 (52.5%)	19 (41.3%)	161 (54.2%)	0.141	29 (37.2%)	15 (38.5%)	14 (35.9%)	0.999
Persistent	163 (47.5%)	27 (58.7%)	136 (45.8%)		49 (62.8%)	24 (61.5%)	25 (64.1%)	
Echocardiographic parameters								
LVEF^a^, % (mean ± SD)	32.7 ± 7.8	37.0 ± 7.1	32.0 ± 7.7	<0.001**	36.1 ± 7.3	36.4 ± 7.3	35.8 ± 7.3	0.667
LVIDd^a^, mm (mean ± SD)	62.7 ± 6.3	60.6 ± 5.0	63.0 ± 6.5	0.035*	61.1 ± 4.5	61.1 ± 5.0	61.2 ± 4.1	0.836
LAD^a^, mm (mean ± SD)	38.4 ± 12.1	45.1 ± 6.0	50.8 ± 9.3	<0.001**	47.1 ± 7.9	45.6 ± 6.2	48.5 ± 9.1	0.114
Medications								
Statin	136 (39.7%)	21 (45.7%)	115 (38.7%)	0.464	37 (47.4%)	18 (46.2%)	19 (48.7%)	0.821
Oral anticoagulant	228 (66.5%)	38 (82.6%)	190 (64.0%)	0.020*	61 (78.2%)	33 (84.6%)	28 (71.8%)	0.273
Antiplatelet	68 (19.8%)	7 (15.2%)	61 (20.5%)	0.520	13 (16.7%)	6 (15.4%)	7 (17.9%)	0.999
Anti-arrhythmics	119 (34.7%)	46 (100.0%)	73 (24.6%)	<0.001**	56 (71.8%)	39 (100.0%)	17 (43.6%)	*p* < 0.001**.
MRA	146 (42.6%)	10 (21.7%)	136 (45.8%)	0.004*	27 (34.6%)	10 (25.6%)	17 (43.6%)	0.153
Digoxin	15 (4.4%)	0 (0.0%)	15 (5.1%)	0.003*	5 (6.4%)	0 (0.0%)	5 (12.8%)	0.055
Beta blocker	207 (60.4%)	23 (50.0%)	184 (62.0%)	0.168	41 (52.6%)	21 (53.8%)	20 (51.3%)	0.821
Non-dihydropyridine CCB	26 (7.6%)	1 (2.2%)	25 (8.4%)	0.227	6 (7.7%)	1 (2.6%)	5 (12.8%)	0.200
ACE-I/ARB	67 (19.5%)	15 (32.6%)	52 (17.5%)	0.028*	25 (32.1%)	13 (33.3%)	12 (30.8%)	0.999
Sacubitril + Valsartan	118 (34.4%)	11 (23.9%)	107 (36.0%)	0.149	25 (32.1%)	10 (25.6%)	15 (38.5%)	0.332
SGLT2 inhibitor	58 (16.9%)	8 (17.4%)	50 (16.8%)	0.999	11 (14.1%)	8 (20.5%)	3 (7.7%)	0.193

PSM, propensity score matching; SD, standard deviation; bpm, beats per minute; LVEF, left ventricular ejection fraction; LVIDd, left ventricular internal dimension in diastole; LAD, left atrium diameter; AF, atrial fibrillation; SAPT, single antiplatelet therapy; NOAC, novel oral anticoagulant; MRA, mineralocorticoid receptor antagonist; CCB, calcium channel blocker; ACE-I, angiotensin-converting-enzyme inhibitor; ARB, angiotensin receptor blocker; SGLT2, sodium-glucose cotransporter-2.

^a^ Mann–Whitney test, the rest were two-way Chi-square tests or Fisher's exact tests.

* Significant with *p *< 0.05.

** Highly significant with *p *< 0.001.

PVI was successfully performed in all patients in Group 1. CTI ablation, linear ablation and substrate modification were performed in 73.9% (*n* = 34), 8.7% (*n* = 4) and 23.9% (*n* = 11) of patients, respectively. Non-PV triggers were identified in 8.7% (*n* = 4) of patients. Notably, the non-PV triggers were from the superior vena cava (75%, *n* = 3), LA anterior wall (50%, *n* = 2), and LA roof (25%, *n* = 1).

The overall recurrence rate of any atrial arrhythmia (AF or AT) in Group 1 patients was 30.4% (*n* = 14) after a mean follow-up duration of 7.7 years after CA. Of note, 21.1% (*n* = 4) of the patients with paroxysmal AF and 37.0% (*n* = 10) of the patients with persistent AF developed recurrence. Among the 14 patients with recurrence, 85.8% (*n* = 12) had recurrent AF, 7.1% (*n* = 1) had AT recurrence, and 7.1% (*n* = 1) had atypical atrial flutter recurrence. Of the patients with recurrence, five (35.7%) underwent repeat CA. During the repeat procedures, the cause of recurrence was PV reconnection in 3 patients (60%), non-PV triggers (LA atrial appendage base and superior vena cava) in 2 (40%), and atypical flutter (perimitral and RA free wall reentry) in 1 patient (20%).

No major adverse events were reported in Group 1. Femoral hematoma developed in 4.3% (*n* = 2) of the patients and 2.2% (*n* = 1) had pericardial effusion. All patients were managed conservatively. In addition, 2.2% (*n* = 1) of the patients developed acute HF with pulmonary edema, which resolved after diuretic administration. All the patients who developed complications were discharged after the procedure.

#### 3.1.2 Predictors of AF recurrence

The baseline characteristics, comorbidities, echocardiographic parameters, and ablation modalities were analyzed to predict AF recurrence. The LAD was the only predictor of AF recurrence after a single procedure (HR, 1.179; 95% CI 1.022 to 1.359, *p* = .024) in univariate Cox regression (Table 2).

**Table 2:** Univariable Cox proportional hazard regression.

	Univariable analysis (AF recurrence)		
	HR	95% C.I.	*p* value
Age, year^a^	0.996	0.931–1.066	0.913
Sex			
Male	Reference	–	0.781
Female	0.740	0.088–6.204	
Heart rate, bpm^a^	0.997	0.967–1.027	0.824
LVEF, %^a^	0.935	0.860–1.016	0.114
LVIDd, mm	1.072	0.910–1.262	0.406
LAD, mm^a^	1.179	1.022–1.359	0.024*
Underlying diabetes			
No	Reference		
Yes	0.046	0.001–235,431.074	0.695
CHA_2_DS_2_VAS_c_ (mean ± SD)			
1	Reference	–	–
2	0.383	0.043–3.458	0.393
≥3	0.819	0.150–4.486	0.818
Types of AF			
Paroxysmal	Reference	–	
Persistent	1.823	0.353–9.418	0.474

LVEF, left ventricular ejection fraction; LVIDd, left ventricular internal dimension in diastole; LAD, left atrium diameter.

^a^ Continuous variables, with risk for each one unit increase.

* Significant with *p *< 0.05.

#### 3.1.3 Part I study: subgroup analysis of LVEF ≤ 30%

A total of 8 patients (17.4%) in Group 1 had LVEF ≤ 30% prior to ablation. Atrial arrhythmia recurrence occurred in four patients (50%). The types of recurrence included AF (75%, *n* = 3) and AT (25%, *n* = 1). In this group, 50% (*n* = 2) of patients underwent repeat procedures. The causes of recurrence included PV reconnection (*n* = 1, 50%) and perimitral flutter (*n* = 1, 50%). There was no significant difference in recurrence between the patients with LVEF > 30% and ≤30% (28.3% vs. 50%, *p* = .418).

### 3.2 Part II study

#### 3.2.1 Baseline characteristics

Before PS matching, there were significant differences in the baseline characteristics between Group 1 and Group 2. Group 1 had younger patients, more men, fewer individuals with diabetes and lower CHA_2_DS_2_VASc scores. Group 1 patients also had higher LVEF, lower LVIDd, lower LAD, more anticoagulants, antiarrhythmic drugs, and ACEi/ARB, and fewer mineralocorticoid receptor antagonists and digoxin. After PS matching, there was no significant difference between PS-Group 1 and PS-Group 2, except that antiarrhythmic drugs were prescribed more frequently in Group 1 (*p* < .001) (Table 1).

#### 3.2.2 Primary and secondary outcome

The overall mortality rate was 20.1% (*n* = 69). The causes of death included cardiovascular-related death in 69.6% (*n* = 48), sepsis-related death in 26.2% (*n* = 18), ischemic stroke in 1.4% (*n* = 1), 1.4% (*n* = 1) pulmonary embolism, and 1.4% (*n* = 1) upper gastrointestinal bleeding.

**Figure 2:**
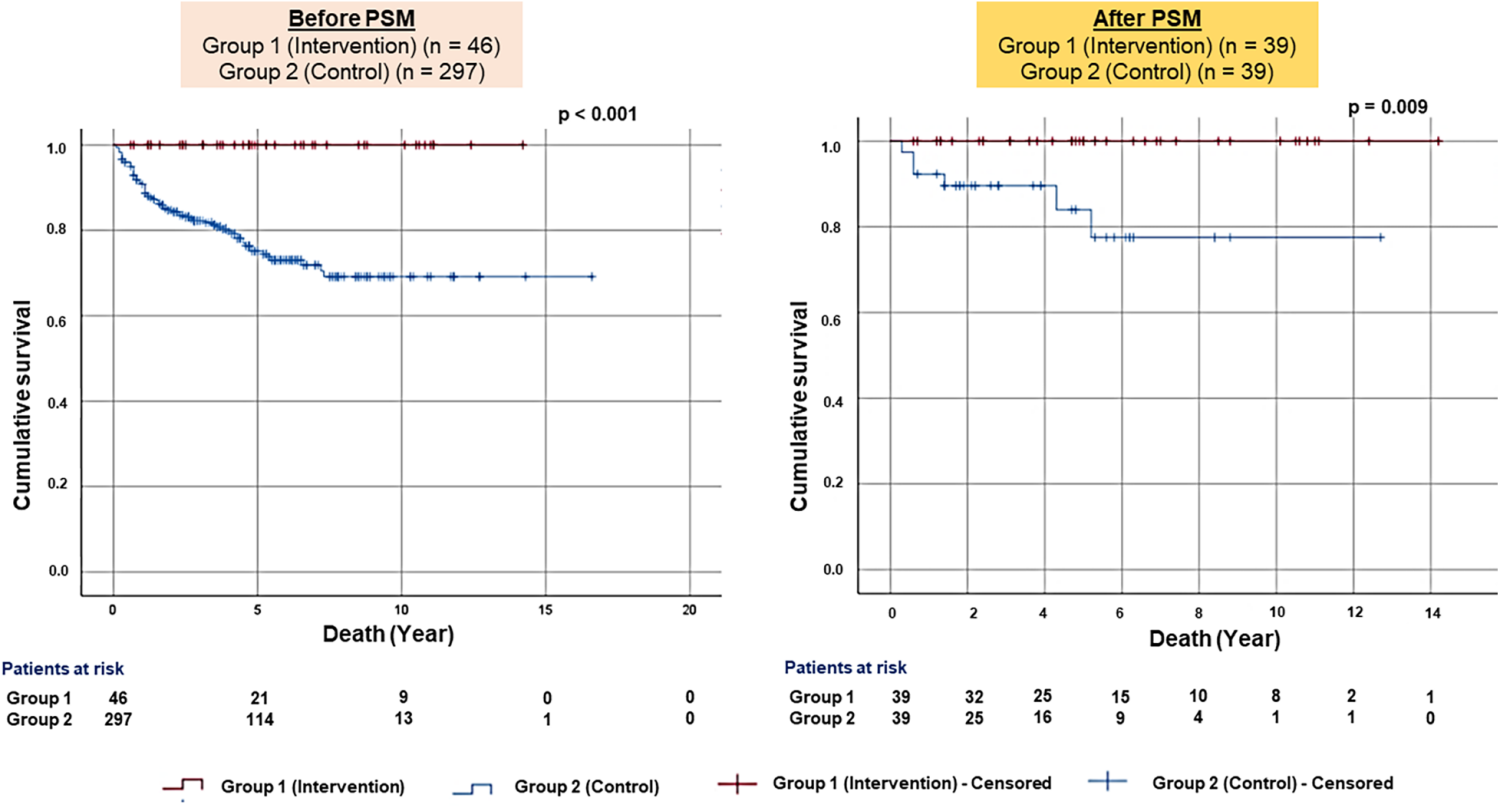
Kaplan–Meier survival plots for all-cause mortality. The figure presented the Kaplan–Meier survival plots for all-cause mortality before (left panel) and after the PS-matched (right panel) patients.

Kaplan–Meier analysis of survival from all-cause mortality showed significantly better survival in Group 1 than in Group 2 before (*p* < .001) and after PS matching (*p* = .009) (Figure 2).

**Figure 3:**
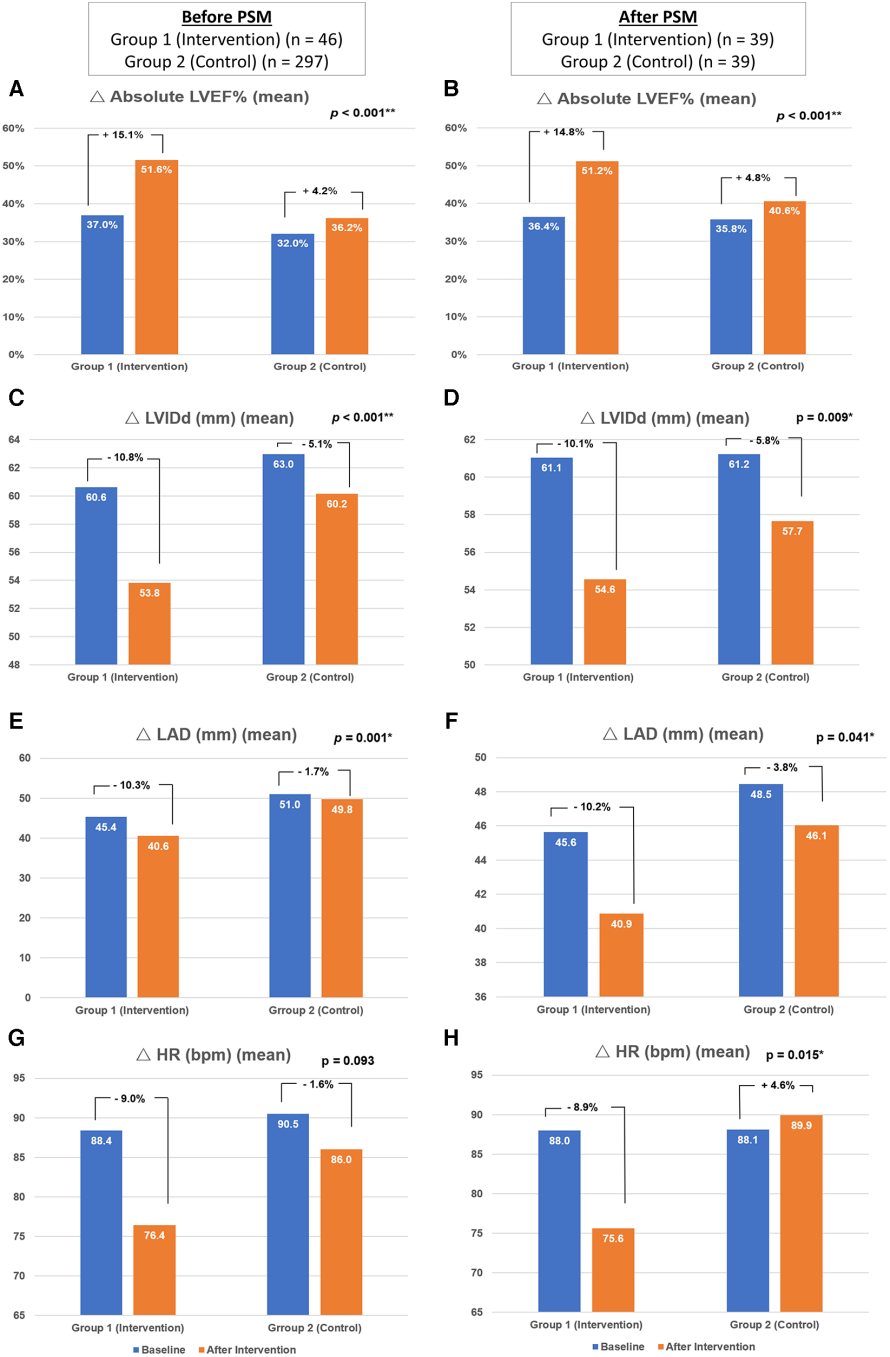
Changes in the echocardiographic parameters and heart rate. The figure demonstrated the changes of absolute left ventricle ejection fraction (**A**,**B**), left ventricular internal diameter in diastole (**C**,**D**), left atrium diameter (**E**,**F**) and heart rate (**G**,**H**) of respondents before (*n* = 343) and after (*n* = 39) propensity score matching. PSM, indicates propensity score matching; LVEF, left ventricular ejection fraction; LVIDd, left ventricular internal dimension in diastole; LAD, left atrium diameter, bpm: beats per minute.

The heart rate was decreased in both Group 1 and Group 2 without significant difference before PS matching (reduction of heart rate: −9.0 ± 21.2% vs. −1.6 ± 26.8%, *p* = .093). After PS matching, there was a statistically higher reduction of heart rate in PS-Group 1 compared to PS-Group 2 (−8.9 ± 22.9% vs. 4.6 ± 24.7%, *p* = 0.015) (Figures 3g,h; Table 3).

**Table 3:** Primary and secondary outcomes before and after propensity score matching.

	Before PSM, *n* (%)			*p* value	After PSM, *n* (%)			*p* value
	Total (*n* = 343)	Group 1 (Intervention) (*n* = 46)	Group 2 (Control) (*n* = 297)		Total (*n* = 78)	Group 1 (Intervention) arm (*n* = 39)	Group 2 (Control) (*n* = 39)	
Outcomes								
Primary outcome: All-cause mortality	69 (20.1%)	0 (0.0%)	69 (23.2%)	0.001*	6 (7.7%)	0 (0.0%)	6 (15.4%)	0.025*
Secondary outcome: MACE								
Cardiovascular mortality	48 (14.0%)	0 (0.0%)	48 (16.2%)	0.007*	3 (3.8%)	0 (0.0%)	3 (7.7%)	0.240
Ischemic stroke	17 (5.0%)	1 (2.2%)	16 (5.4%)	0.709	3 (3.8%)	0 (0.0%)	3 (7.7%)	0.240
Myocardial infarction	7 (2.0%)	0 (0.0%)	7 (2.4%)	0.601	0 (0.0%)	0 (0.0%)	0 (0.0%)	NA
Hospitalization due to heart failure	105 (30.6%)	1 (2.2%)	104 (35.0%)	<0.001**	10 (12.8%)	1 (2.6%)	9 (23.1%)	0.018*
% Changes of heart rate, mean ± SD^a^	−2.6 ± 26.2	−9.0 ± 21.2	−1.6 ± 26.8	0.093	−2.1 ± 24.6	−8.9 ± 22.9	4.6 ± 24.7	0.015*
Echocardiographic parameters								
Changes of absolute LVEF, %^b^, mean ± SD	5.8 ± 11.3	15.1 ± 10.9	4.2 ± 10.6	<0.001**	9.8 ± 11.7	14.8 ± 11.2	4.8 ± 10.2	<0.001**
% Changes of LVIDd^b^, mean ± SD	−6.2 ± 13.5	−10.8 ± 12.5	−5.1 ± 12.2	<0.001**	−8.0 ± 10.9	−10.1 ± 11.8	−5.8 ± 9.7	0.009*
% Changes of LAD^b^, mean ± SD	−4.5 ± 14.3	−10.3 ± 13.3	−1.7 ± 14.0	0.001*	−7.0 ± 14.1	−10.2 ± 12.5	−3.8 ± 14.9	0.041*

PSM, propensity score matching; MACE, major adverse cardiovascular events; LVEF, left ventricular ejection fraction; LVIDd, left ventricular internal dimension in diastole; LAD, left atrium diameter; SD, standard deviation.

^a^ Before PSM: Mann–Whitney test, after PSM: Student's t test.

^b^ Mann–Whitney test, the rest were two-way Chi-square tests or Fisher's exact tests.

* Significant with *p *< 0.05.

** Highly significant with *p *< 0.001.

As for the secondary outcome, the cardiovascular mortality rate was 14% (*n* = 48) and the incidence of HF hospitalization was 30.6% (*n* = 105) across all patients in Groups 1 and 2. Before PS matching, Group 1 patients exhibited a lower incidence of cardiovascular mortality [Group 1 vs. Group 2: 0% (*n* = 0) vs. 16.2% (*n* = 48), *p* = .007] and HF hospitalization [Group 1 vs. Group 2: 2.2% (*n* = 1) vs. 35.0% (*n* = 104), *p* < .001]. After PS matching, there were still fewer HF hospitalizations in PS-Group 1 than in PS-Group 2 patients [PS-Group 1 vs. PS-Group 2: 2.6% (*n* = 1) vs. 23.1% (*n* = 9), *p* = .018]. There were no significant differences in the incidences of ischemic stroke or myocardial infarction between the two groups (Table 3).

Regarding the echocardiographic parameters, there was a significant improvement in Group 1 compared to Group 2. Before PS matching, there was a better improvement of absolute LVEF in Group 1 than in Group 2 (15.1 ± 10.9% vs. 4.2 ± 10.6%, *p* < .001). The reduction of LVIDd (−10.8 ± 12.5% vs. −5.1 ± 12.2%, *p* < .001) and LAD (−10.3 ± 13.3% vs. −1.7 ± 14.0%, *p* = .001) were also more significant in Group 1 than in Group 2. After PS matching, the increment of absolute LVEF remained more significant in PS-Group 1 than in PS-Group 2 (14.8 ± 11.2% vs. 4.8 ± 10.2%, *p* < .001). The reduction of LVIDd (−10.1 ± 11.8% vs. −5.8 ± 9.7%, *p* = .009) and LAD (−10.2 ± 12.5% vs. −3.8 ± 14.9%, *p* = .041) were also more significant in PS-Group 1 compared to PS-Group 2 (Table 3; Figures 3a–f).

#### 3.2.3 Subgroup analysis LVEF ≤ 30%

In the subgroup analysis of the patients with LVEF ≤ 30% (8 patients in Group 1 and 121 patients in Group 2), there was less incidence of hospitalization due to HF in Group 1 when compared with Group 2 [0% (*n* = 0) vs. 43.8% (*n* = 53), *p* = .021]. There were no significant differences in the overall mortality, cardiovascular mortality, ischemic stroke, or myocardial infarction in this subgroup analysis.

## 4 Discussion

### 4.1 Main findings

The present study has several main findings. First, CA for AF in patients with DCM can achieve a good AF-freedom outcome (30.4% recurrence rate) in long-term follow-up. Second, the LAD was the only factor that predicted AF recurrence in patients with DCM. Third, CA significantly improved clinical outcomes and reversed structural remodeling in both LA and LV.

### 4.2 Recurrence of AF in DCM patients after CA

The overall AF recurrence rate in patients with DCM after CA was 30.4%. In previous studies with ten-year follow-up, the recurrence of AF after the index AF procedure was 42% and 84% in patients with paroxysmal and persistent AF, respectively (21, 22). The recurrence rate was lower in the CA group than in the medical therapy group. This finding requires further studies with a larger number of patients. Additionally, PV reconnection is a major cause of AF recurrence after repeated procedures. This result is in line with those of previous studies (21, 22) as the major triggers of PV in both paroxysmal and persistent AF (23). The results of the present study highlighted the benefits of CA in patients with DCM and AF. Further prospective studies are required to determine the advantages of CA in this cohort.

### 4.3 Predictor of recurrence in DCM patients after CA

In the present study, LAD was the only predictor of atrial tachyarrhythmia recurrence in patients with DCM. This result is consistent with those of previous studies (21, 22, 24). LA dilatation is more than just an increment in size, it is also a manifestation of LA fibrosis and remodeling. These are the substrates for AF occurrence, and advanced stages of fibrosis and remodeling are also considered important factors in predicting recurrence after catheter ablation (25–28).

### 4.4 Survival in DCM patients after CA

The prevalence of DCM with AF in our registry was 4.0%, similar to real-world data of 5.9% by Buckley et al. (29) To the best of our knowledge, this study is the first to demonstrate improvements in survival rates and overall echocardiographic parameters among patients with AF and DCM after receiving CA compared to medical therapy, at a mean follow-up of 7.7 years.

Compared to anti-arrhythmic drugs, CA was proven to reduce mortality and HF hospitalization in HF patients. Previous landmark studies include AATAC, Castle-AF and Castle-HTx delineated the superiority of CA while compared to medical therapy in AF and HF patients (9, 10, 30). However, these studies did not address DCM in particular.

Furthermore, Zhao et al. demonstrated that CA improved heart failure in DCM and AF patients during the early stages, but this improvement was not sustained beyond three years (31). Another study by Rillig et al. showed improvements in LVEF and a reduction in LAD among AF and DCM patients who received CA, but statistically not significant in the reduction of LVIDd (32). In the same study, a higher mortality rate was observed in the DCM group compared to those with arrhythmia-induced cardiomyopathy. Thus, in the current study, CA significantly reduced all-cause mortality, cardiovascular death, and HF hospitalization in patients with DCM and AF compared with those treated with medication alone.

The outcomes of DCM have been poor in the past. A previous study demonstrated that ACEi/ARB and beta-blockers improved the survival rates of patients with DCM from 55% to 87% over a mean follow-up period of 8 years (33). The survival rate with medical therapy was 79.9% in the current group of patients with DCM and AF, which was comparable to that reported in a previous study (8, 12). Further prospective randomized studies were required to prove the benefit of AF ablation regarding the clinical outcome in DCM patients.

### 4.5 Benefit of combination of CA and medication in DCM with AF patients

The present study demonstrated a significant increase in LVEF and a reduction in both LVIDd and LAD in patients with DCM after CA for AF. LVEF improvement was in line with the previous studies (10, 11, 30). As mentioned previously, GDMT has been proven to improve long-term outcomes and reduce mortality in patients with DCM (33, 34).

After PS matching, there were no discernible differences in the usage of oral anticoagulants and SGLT2 inhibitors between patients receiving CA and medical therapy. However, clinicians may incline to administer more intensive treatments to patients in the CA patients, which could potentially impact the long-term outcomes.

In addition, heart rate strongly predicts cardiovascular outcomes in patients with DCM (34). However, optimal rate control using medication alone for DCM remains a challenge. In this study, there was a significant reduction in heart rate in patients with CA compared to those receiving medication only. Therefore, in the present study, adjuvant CA in addition to GDMT improved structural remodeling and long-term outcomes.

### 4.6 Limitations

This study has several noteworthy limitations. Since data were retrospectively collected from a hospital electronic database, some comorbidities may have been underreported. Second, this single-center study conducted in Taiwan may not represent the general population with DCM and AF. Third, relatively small sample size in the patients receiving CA and a selection bias might have existed despite with PS matching because of the retrospective nature of the study. Although the PS matching method might adjust the differences in patient characteristics, but could not eliminate the selection bias, especially considering procedure-related outcomes. Fourth, advancement in AF ablation technologies and emergence of new medications such as sacubitril/valsartan or SGLT2 inhibitor may affect the outcomes in this very long-term follow up populations. Further studies with a standardized protocol for medication and ablation strategies are warranted. Fifth, patients who received CA may be subjected to more intensive monitoring, integrated care and treatment. These strategies might translate into a better outcome in CA arm. Lastly, strict screening and protocols such as genetic screening, endomyocardial biopsy or cardiac magnetic resonance imaging were not mandatory for all patients. This may affect the result of the study.

## 5 Conclusion

In addition to conventional HF treatment, CA is effective for long-term AF and HF control in patients with DCM. Compared to medical therapy, adjuvant CA significantly improves overall survival, reduces cardiovascular mortality, and improves structural remodeling in the LA and LV in patients with AF and DCM.

## Data Availability

The original contributions presented in the study are included in the article/Supplementary Material, further inquiries can be directed to the corresponding author.
